# Collagen-disruptive cell therapy: adoptive transfer of membrane-anchored, tumor cell surface vimentin-targeted interleukin 12–armed TILs suppress collagen expression to boost deep T-cell infiltration via dual signaling activation and significant CCKAR reduction

**DOI:** 10.21203/rs.3.rs-5104493/v1

**Published:** 2024-10-29

**Authors:** Shulin Li

**Affiliations:** The University of Texas MD Anderson Cancer Center

## Abstract

Tumor-targeted T-cell therapies of various types have been booming, but T-cell therapy is limited by its inability to penetrate the collagen barrier surrounding tumors. The destruction of tumor collagen is significant because collagen both suppresses T cells and contributes to the formation of the extracellular matrix. Our previously reported cell surface vimentin (CSV)–targeted and membrane-anchored IL12-armed (attIL12) T cells can reduce collagen production by killing cancer-associated fibroblasts, thereby increasing T-cell infiltration. However, attIL12-T cells cannot reduce collagen expression by tumors that highly express CCKAR. In this study, we discovered that CCKAR directly boosts collagen production by tumor cells in vitro and in vivo. attIL12-modified tumor-infiltrating lymphocytes (TILs) disabled collagen production by CCKAR-high autologous tumor cells in vitro and sarcoma patient-derived xenografts (PDXs) in vivo. This disruption of collagen production by tumor cells required a simultaneous interaction between the CSV on autologous tumor cells, which is targeted by attIL12, and HLA-TCR on attIL12-TILs; when either interaction was abrogated, collagen production and CCKAR expression were not shut down. Mechanistically, the interaction between attIL12-TILs and autologous tumor cells synergized IFNγ production, which in combination with CCKAR downregulation reduced collagen expression through suppression of both TGFβ-stimulated SMAD activation and CCKAR-AKT signaling. Diminishing collagen expression from tumor cells significantly increased T-cell infiltration and improved tumor growth inhibition in PDX sarcomas. This study thus uncovers the first tumor collagen–disrupting T-cell therapy we know of. This is significant because collagen is enriched in most high-grade CCKAR+ human sarcomas. Thus, this attIL12-TIL therapy holds great clinical potential for boosting T-cell infiltration in high-grade, collagen-rich tumors.

## Introduction

Sarcoma is a rare cancer that can occur in childhood (osteosarcoma) or adulthood (undifferentiated sarcoma). The standard treatment for sarcoma is surgical removal along with radiation therapy and chemotherapy, but sarcoma tumors often recur and metastasize. Thus, there is an urgent need for novel and effective treatment approaches. Immunotherapy has attracted growing interest for treating sarcoma. Among current cancer immunotherapies, adoptive transfer of tumor-infiltrating lymphocytes (TILs) has shown advantages over other T-cell therapies for treating solid tumors, with consistent objective response rates of 70% and complete remission rates of up to 20% in several melanoma clinical trials.^1^ Clinical trials to investigate the safety and efficacy of TIL therapy in other advanced solid tumors are also under way.^2, 3^ The first clinical trial of adoptive TIL therapy for sarcoma has launched and has shown the feasibility of this treatment for sarcoma patients.^4^ However, a major challenge of TIL therapy in clinical settings is the inability of TILs to penetrate tumors after *ex vivo* expansion.

A big hurdle for the access of adoptively transferred immune cells to sarcoma cells is that these tumors exhibit mesenchymal differentiation, large deposits of extracellular matrix (ECM), and high rigidity ^5–7^. The ECM richness of sarcoma tumors may create a collagen-based protective barrier around the tumors that excludes immune cells.^8, 9^ There are currently no effective approaches to reduce collagen density in tumor stroma, that is, to loosen the tight connection between cells within a tumor and make them accessible to cytotoxic T cells. This study aimed to address this challenge.

Expression of collagen genes (e.g., *COL1A1* and *COL1A2*) is induced in tumor stromal cells such as cancer-associated fibroblasts (CAFs) and in tumor cells by transforming growth factor beta 1 (TGFβ1)-mediated phosphorylated SMAD3 (pSMAD3) signaling. This signaling pathway is activated upon the transition of a latent form of TGFβ1 to an active form that binds the heteromeric receptor TGFβRI.^10, 11^ Meanwhile, tumor-cell collagen gene expression also requires CCKAR-induced AKT activation, which enhances pSMAD3 signaling and its associated collagen expression.^12^ CCKAR, a G protein-coupled receptor of cholecystokinin, is highly expressed in cancer cells of many types, including gallbladder cancer,^13^ melanoma,^14^ pancreatic cancer,^15^ and non-small cell lung cancer,^16^ and is associated with poor prognosis. In tumor cells, inhibition of AKT activation impairs SMAD3 phosphorylation and in turn abrogates SMAD3-mediated collagen induction, suggesting that crosstalk between AKT signaling and SMAD3 phosphorylation upregulates collagen.^17^ To remove collagen’s obstruction of T-cell infiltration, current collagen-inhibition methods focus on targeting TGFβ1-induced collagen expression, but targeting TGFβ alone via systemic administration of a monoclonal antibody only slightly enhanced T-cell infiltration in humanized tumor models^18^ owing to the constitutively high expression of TGFβ in tumor cells. A more effective approach is urgently needed for the success of T-cell based therapy to treat solid tumors.

Since both TGFβ downstream signaling and CCKAR activation account for collagen expression, a single approach that attenuates both signaling pathways is needed to reduce collagen density in tumors. Given that a high level of interferon gamma (IFNγ) not only counteracts collagen upregulation by impairing SMAD signaling downstream of TGFβ and has been reported to inhibit CCKAR (or CCK1R) expression in chicken cells,^19^ we hypothesized that robust IFNγ elevation in tumors could be the key to diminishing collagen deposition in sarcoma tumors.

Our group previously discovered that cell-surface vimentin (CSV) is exhibited on the surface of a variety of highly malignant solid tumor cells, including sarcoma cells.^20^ It is also well known that wild-type interleukin 12 (IL12) is a potent antitumor agent for the treatment of many types of cancers, but it is notorious for its toxicity to normal tissues.^21^ To reduce the toxicity and increase the tumor specificity of IL12, we discovered a CSV-targeting peptide, VNTANST, and invented a tumor-targeted IL12 (ttIL12), in which a fusion gene encoding this peptide was placed before the stop codon of the IL12 p40 encoding sequence.^22^ We further developed membrane-anchored ttIL12 T cells (attIL12-T cells, using T cells expanded from peripheral blood mononuclear cells) by anchoring ttIL12 to the T-cell surface via a transmembrane domain and demonstrated that these attIL12-T cells produce IFNγ upon binding to CSV on tumor cells.^23, 24^ However, in osteosarcoma patient-derived xenograft (PDX) models, the level of IFNγ induced by the attIL12-CSV interaction inhibited only 50% of tumors with modest levels of collagen and was ineffective in altering collagen-rich models in which high expression of TGFB1 and CCKAR promoted collagen expression.^25^ To reach the threshold level of IFNγ needed in the tumors to reduce dense collagen, here we investigated dual IFNγ-inducing signaling pathways by taking advantage of autologous TIL–PDX tumor pairs to contribute additional IFNγ through T-cell receptor (TCR) activation. We therefore hypothesized that attIL12-transduced TILs synergize two pathways of IFNγ production by enabling the simultaneous interactions of the TCR on TILs and human leukocyte antigen (HLA) on tumor cells and between attIL12 and CSV, destroying the collagen barrier around tumors and opening a channel for T cells to infiltrate and kill tumor cells.

Our findings showed that when engaging with autologous undifferentiated sarcoma and osteosarcoma cells, attIL12-TILs had markedly higher IFNγ production compared to control TILs via dual activation of the attIL12-CSV and TCR-HLA interactions. This synergistic IFNγ elevation suppressed TGFβ-dependent SMAD3 activation and shut down the CCKAR-AKT-SMAD3 pathway, thereby totally abrogating collagen expression both *in vitro* and *in vivo*. Knockdown of either activation mechanism caused a failure to decrease collagen expression in CCKAR+ cells. Collagen impairment in sarcoma tumors resulted in greater T-cell infiltration into the tumor interior and improved the antitumor efficacy of attIL12-TILs in treating autologous sarcoma PDX tumors.

## Results

### attIL12-TILs show better tumor infiltration than control TILs despite similar cell surface marker profiles

Preparation of T cells from patients’ blood is relatively easy, and arming these T cells with attIL12 enhances their infiltration into a small percentage of osteosarcoma PDX tumor models.^23, 24^ However, models with high collagen and rich ECM did not respond to attIL12-T cell treatment.^25^ We found that in these resistant models, the TGFβ-dominated tumor microenvironment promoted ECM development and inhibited IFNγ function.^23, 25, 26^ These resistant tumors also were associated with a high level of CCKAR expression, a biomarker for many cancers that functions as a collagen regulator.^25^ To overcome the rich ECM–associated resistance to attIL12-T cell treatment, we hypothesized that in addition to the engagement between the CSV binding motif on attIL12-T cells and CSV on tumor cells, a second interaction between T cells and tumor cells is required to boost IFNγ elevation and shut down both TGFβ and CCKAR signaling. TILs were an obvious choice for the second interaction because TILs isolated from tumors recognize a variety of tumor-specific antigens and release IFNγ.^25^ To test this hypothesis, we successfully generated 3 independent CSV^+^ PDX tumors: SA127 (undifferentiated sarcoma), SA117 (osteosarcoma), and SA174 (osteosarcoma) *in vivo* and their matched autologous TILs *in vitro*, following a large number of attempts.

**Figure 1:**
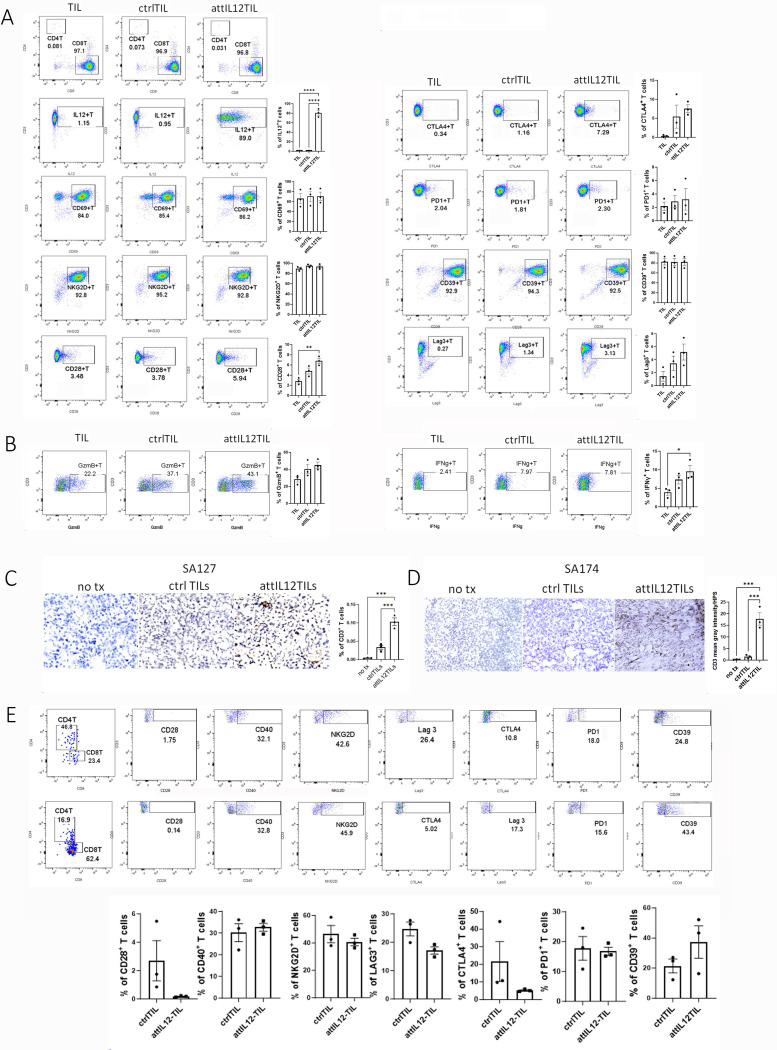
attIL12-TILs show superior tumor infiltration to control TILs despite similar cell surface marker profiles. **(A, B)** Control TILs (ctrl-TILs) and attIL12-TILs show similar cell surface markers and effector molecules *in vitro*. SA127 TILs were transduced with a control vector or attIL12 lentivirus for 48 hours. **(A)** Flow cytometry analysis of cells stained with IL12, CD3, CD4, CD8, CTLA4, PD1, CD28, CD69, LAG3, CD39, and NKG2D. **(B)** Flow cytometry analysis of effector molecules. Cells were treated with brefeldin A 4 hours before harvest. Cells were fixed and permeabilized, followed by staining with IFNγ and granzyme B for flow cytometry analysis. **(C, D)** Representative immunohistochemistry images of T-cell accumulation in PDX tumors. SA127 **(C)** and SA174 **(D)** tumorbearing SCID mice were preconditioned with cyclophosphamide prior to 2 treatments with ctrl-TILs or attIL12-TILs. Two days after the second TIL transfer, tumors were collected, sectioned, and stained with human CD3 via immunohistochemistry. Staining was detected with a Keyence microscope. **(E)** Tumors from (C) were dissociated to single-cell suspensions and stained with the immune cell markers described in (B) for flow cytometry. *P< 0.05; **P < 0.01; ***P < 0.001; ****P < 0.0001.

Our results showed that these TILs were mostly CD8^+^ T cells and that attIL12-modified TILs (attIL12-TILs) highly expressed IL12 on the cell surface (Fig. 1A). When comparing the expression of costimulatory and exhaustion markers on control TILs and attIL12-TILs, we found that attIL12 transduction had little impact on these profiles *in vitro* (Fig. 1A and S1A). We also tested production of cytokines (IFNγ and granzyme B) by the TILs to assess their effector function and found no major differences between control and attIL12-TILs (Fig. 1B, S1B), further revealing that the attIL12 modification does not change the TILs’ characteristics *in vitro*.

Next, we determined whether attIL12-TILs boost infiltration into autologous ECM-rich sarcoma tumors which attIL12-T cells failed to infiltrate.^23, 25^ SA127 or SA174 tumors were transplanted subcutaneously into SCID mice, and control or attIL12-modified autologous TILs were infused intravenously when the tumors reached 6 mm. A second round of TIL infusions was performed 14 days later. Four days after the second TIL infusion, we harvested the tumors to detect human T cells in the tumor sections. Compared to tumors with no treatment, a few control TILs had infiltrated the tumors. However, T-cell accumulation was more than 3-fold higher in the attIL12-TIL–treated tumors (Fig. 1C, 1D). These results demonstrated that control TILs alone are not effective in penetrating PDX tumors (as in human patients), whereas the addition of attIL12 recovered the tumor-infiltrating capacity of TILs in the autologous tumor model. After treatment, we again assessed the cell surface marker profiles of these TILs. This time, attIL12-TILs remained mostly cytotoxic CD8^+^ T cells in contrast to high frequencies of T helper CD4^+^ control TILs (Fig. 1E). Nonetheless, the control and attIL12-TILs showed no significant changes in T-cell marker profiles (Fig. 1E, S1C).

### attIL12-TIL treatment boosts antitumor efficacy against autologous tumors

**Figure 2:**
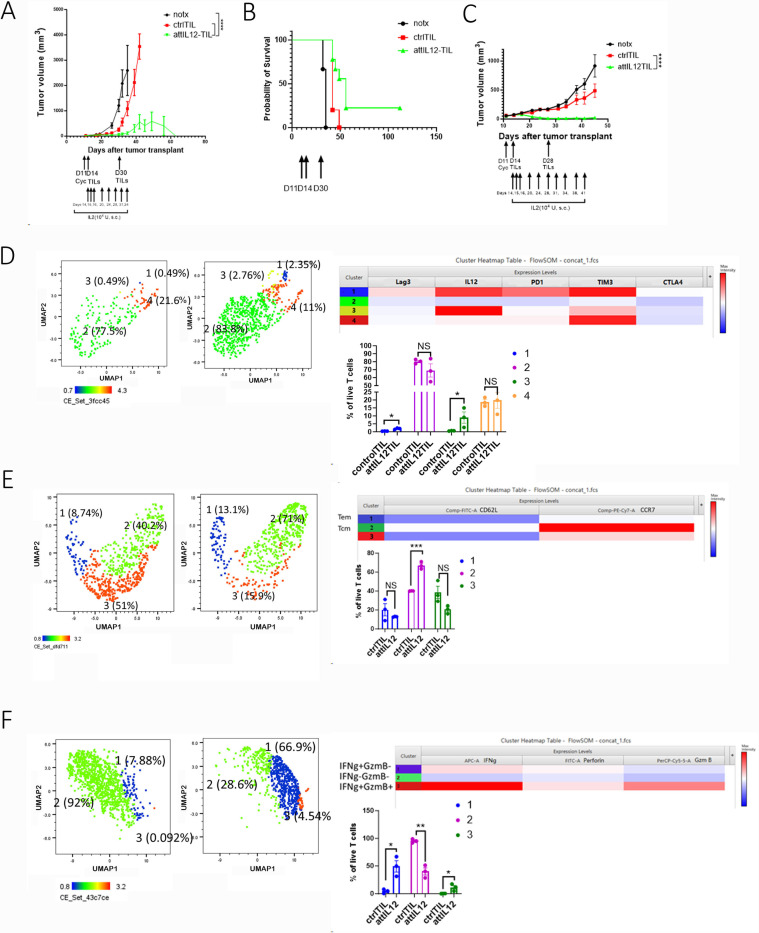
attIL12-TIL treatment boosts antitumor efficacy against autologous tumors compared to control-TILs. **(A, C)** Growth curves for SA127 **(A)** and SA174 **(C)** tumors. Tumor-bearing SCID mice were preconditioned with cyclophosphamide (Cyc) prior to 2 treatments with ctrl-TILs or attIL12-TILs on the indicated days (black arrows). Tumor volume was monitored twice weekly. **(B)** Kaplan-Meier curves comparing overall survival time in mice bearing SA127 tumors. **(D-F)** UMAP dimension reduction (left), heatmaps (top right), and graphs (bottom right) show phenotypic clusters of each treatment group. Mice from (A) were treated with brefeldin A 4 hours prior to tumor collection. Tumors were dissociated to single-cell suspensions, stained with the indicated cell surface markers, memory markers, and effector molecule markers, and subjected to flow cytometry. *P < 0.05; **P< 0.01; ***P < 0.001; ****P < 0.0001; NS: not significant.

High T-cell infiltration is a predictor of immunotherapy response in solid tumors^27^; this is also true of our attIL12-TIL therapy. After 2 infusions of attIL12-TILs, the autologous sarcoma PDX tumors regressed in the SA127 (Fig. 2A) and SA174 models (Fig. 2C), resulting in 2 of 9 mice becoming tumor free in SA127 and 3 of 5 mice becoming tumor free in SA174 (Fig. 2B, 2C). No long-term adverse effects were observed in the tumor-free survivors. Control TILs temporarily delayed, but failed to inhibit tumor progression (Fig. 2A–2C).

The causes of this significant difference in antitumor efficacy could be differences in the TILs’ cytolytic activity, their tumor-infiltration capacity, or both. To determine which mechanism plays the major role, we dissociated the tumors and assessed the expression of cell surface markers, cell memory markers, and effector molecules on control and attIL12-TILs using flow cytometry (Fig. 2D–2F, S2, S3). Unsupervised clustering analysis of the cell surface markers on all live CD45+ T cells in SA127 tumors revealed 4 clusters (1: IL12 (membrane bound) ^+^PD1^+^TIM3^+^; 2: IL12^−^; 3: IL12^+^TIM3^lo^; and 4: IL12^−^TIM3^hi^). Distinct uniform manifold approximation and projection (UMAP) analysis of these cells showed that attIL12-TILs exhibited dramatically higher cell membrane IL12 expression than did control TILs (Fig. 2D, clusters 1 and 3). The T-cell memory marker analysis identified 3 clusters in which attIL12-TILs exhibited enhanced effector memory and central memory markers over control TILs (Fig. 2E, cluster 1 vs. cluster 2). Finally, for effector molecule production, attIL12-TILs showed markedly greater abundance of cluster 1 (IFNγ^+^GzmB^−^) and cluster 3 (IFNγ^+^GzmB^+^), whereas control TILs were predominantly classified as cluster 2 (IFNγ^−^GzmB^−^) (Fig. 2F). Similar features of TILs were observed in the SA174 PDX model after control TIL or attIL12-TIL transfer (Fig. S3). These results collectively suggested that the cytolytic activities of control TILs were largely suppressed after tumor infiltration, possibly owing to a TGFβ-dominated tumor microenvironment. In striking contrast, attIL12-TILs promoted IFNγ production when interacting with autologous tumor cells to overcome immune suppression and maintain antitumor cytotoxicity.

### High collagen deposition in human sarcoma tissues is associated with advanced tumor stage

**Figure 3:**
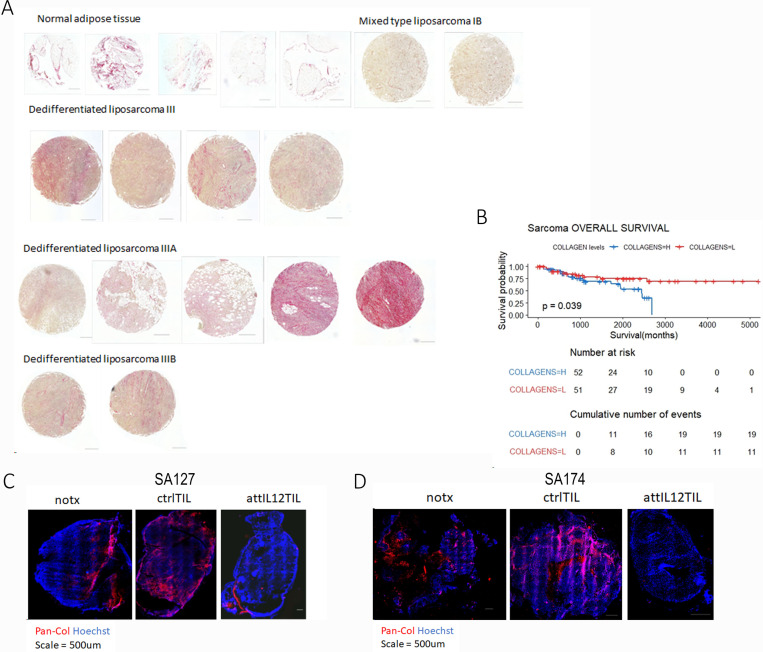
High collagen deposition in human sarcoma tissues is associated with shorter survival. **(A)** Representative images of Liposarcoma tissue microarrays of stages were stained with Sirius red to identify collagen density. **(B)** Kaplan-Meier curves showing association of survival time with pan-collagen gene expression in TCGA-SARC dataset. Collagens=H indicates top 20% of patients by pan-collagen gene expression; Collagens=L indicates bottom 20%. **(C,D)** Representative immunofluorescence staining showing pan-collagen density in SA127 **(C)** and SA174 **(D)** tumors with no treatment, treatment with ctrl-TILs, or treatment with attIL12-TILs. Scale bar: 500μm.

Another cause of the differences in the effectiveness of control TIL and attIL12-TIL treatments may be tumor infiltration efficiency, which can be altered by the density of collagen in tumors. We focused on collagen because *CCKAR*, the most upregulated gene in sarcoma models that were resistant to attIL12-T cell treatment, is associated with collagen production, which may form immune-blocking walls to facilitate tumor development. To determine the collagen deposition in sarcoma tumors, we used a tissue microarray containing 5 normal adipose tissues, 2 stage I malignant liposarcoma clinical samples, 4 stage II samples, and 7 stage III samples. Sirius red staining showed very low levels of collagen in the normal and stage I liposarcoma tissues, and a much higher density of collagen deposition in the stage III tissues (Fig. 3A). Such high collagen density was significantly associated with poor overall survival in sarcoma patients, as determined by a survival analysis of the sarcoma dataset from The Cancer Genome Atlas (TCGA) (Fig. 3B). Patients were stratified by *COL1A1*, *COL1A2*, *COL2A1*, and *COL6A1* expression, and survival in the 20% of patients with the highest expression of these genes was compared with that of the 20% of patients with the lowest expression. The log-rank (Mantel-Cox) test showed that patients with high levels of these pan-collagen genes had shorter overall survival time than did patients with low pan-collagen expression (*P* = 0.039) (Fig. 3B).

To determine if collagen density plays a critical role in PDX tumors, as it did in the clinical samples, we assessed the collagen density in SA127 (Fig. 3C) and SA174 (Fig. 3D) tumors after control TIL or attIL12-TIL treatment via whole tumor section scans of immunofluorescence-stained collagen. Our results showed that attIL12-TIL treatment almost eliminated collagen expression in the autologous tumors while control TIL treatment did not reduce collagen expression (Fig. 3C, 3D).

### attIL12-TILs induce robust IFNγ expression to diminish collagen production by sarcoma cells

In tumors, collagens and other ECM factors involved in tumor microenvironment remodeling are mainly produced and regulated by stromal cells (e.g., CAFs).^28^ We previously demonstrated that attIL12-T cell therapy induces IFNγ in CSV^+^ PDX tumors, causing apoptosis of CAFs and leading to the destruction of the collagen structure in osteosarcoma tumors with modest ECM.^23, 25, 29^ Recent reports showed evidence that tumor cells also produce collagens that play crucial roles in cancer development.^30, 31^ To determine which cell type is the primary source of collagen expression in our collagen-rich, attIL12-T cell–resistant sarcoma PDX models, we first identified MDM2 and B7H3 as the sarcoma markers of SA127 and SA174, respectively (Fig. S4A, S4B). From dissociated tumors, collagen expression was predominantly from MDM2^+^ (SA127) or B7H3^+^ (SA174) tumor cells (Fig. S4C, S4D), suggesting that tumor cells serve as the primary source of collagens in these PDX models. In the liposarcoma PDX model YN20, compared to attIL12-T cells, which impaired CAFs, attIL12-TILs reduced both CAFs and tumor cell–derived collagen (Fig. S4E). Since disruption of CAFs alone by attIL12-T cell treatment failed to overcome ECM-rich sarcomas,^23^ our mechanistic study next focused on reducing the collagen production by autologous tumor cells. Compared to attIL12-T cells and control-TILs, only attIL12-TIL coculture reduced collagen expression in YN20 sarcoma PDX tumors (Fig. S4E).

**Figure 4:**
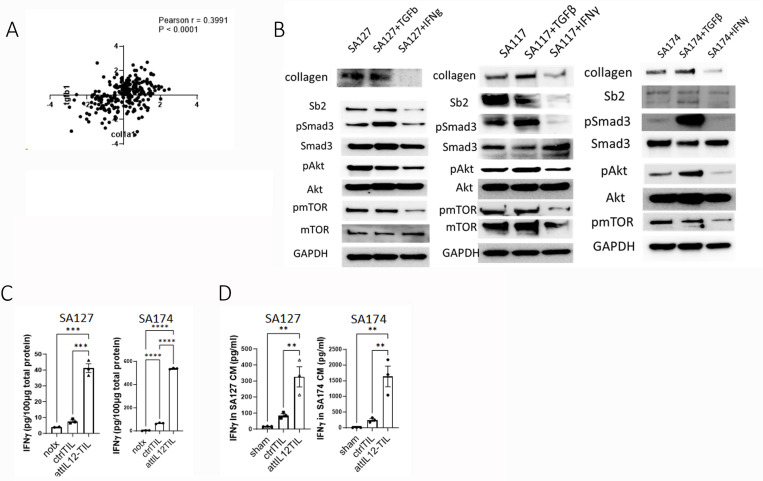
A shift in the balance of cytokines regulates collagen production by sarcoma cells. **(A)** Pearson correlation analysis of TCGA sarcoma patient dataset analysis of *TGFB1*and *COL1A1* mRNA expression. **(B)** Representative immunoblots showing collagen, SMAD signaling, and AKT signaling expression in SA127, SA117, and SA174 tumor cells treated with TGFβ (100 ng/mL) or IFNγ (100 ng/mL) for 24 h. **(C)** ELISA results showing IFNγ levels in tumors treated as indicated. **(D)**ELISA results showing IFNγ levels in supernatant from coculture of autologous tumor cells with control-TILs or attIL12-TILs. *P < 0.05; **P< 0.01; ***P < 0.001; ****P < 0.0001.

TGFβ is known to regulate collagen expression. Analysis of the TCGA sarcoma dataset suggested that *TGFB1* mRNA expression (z-score) was positively correlated with *COL1A1* expression (*P* < 0.0001, Pearson *r*: 0.3991) (Fig. 4A). We next used immunoblotting to examine whether a switch in the balance of TGFβ and IFNγ levels also affected collagen expression in our sarcoma PDX cells. SA127 and SA174 tumor cells were treated with TGFβ or IFNγ. While TGFβ treatment increased collagen expression, IFNγ completely abolished it. Activation of SMAD3 and AKT, which is triggered by TGFβ signaling to induce collagen expression, was also inhibited by IFNγ in these tumor cells (Fig. 4B), suggesting that abundant IFNγ reduces TGFβ-mediated collagen expression in sarcoma cells. If IFNγ elevation is the true mechanism behind the reduction of collagen density in collagen-rich sarcomas, we should detect a robust IFNγ increase after attIL12-TIL treatment. In SA127 and SA174 tumors, IFNγ levels were indeed significantly higher in the attIL12-TIL–treated tumors compared to the untreated or control-TIL–treated ones (Fig. 4C).

To further validate the robust IFNγ production by attIL12-TILs, we set up an *in vitro* model by coculturing SA127 or SA174 tumor cells with autologous control or attIL12-TILs at a tumor cell:T cell ratio of 4:1 for 24 hours. The supernatant was collected for ELISA, which also showed significant IFNγ induction after attIL12-TIL coculture (Fig. 4D), in line with the *in vivo* results (Fig. 4C). One remaining question is *how* attIL12-TILs were stimulated to robustly induce IFNγ and reduce collagen.

### Dual signaling activation is essential for the potency of attIL12-TILs

**Figure 5:**
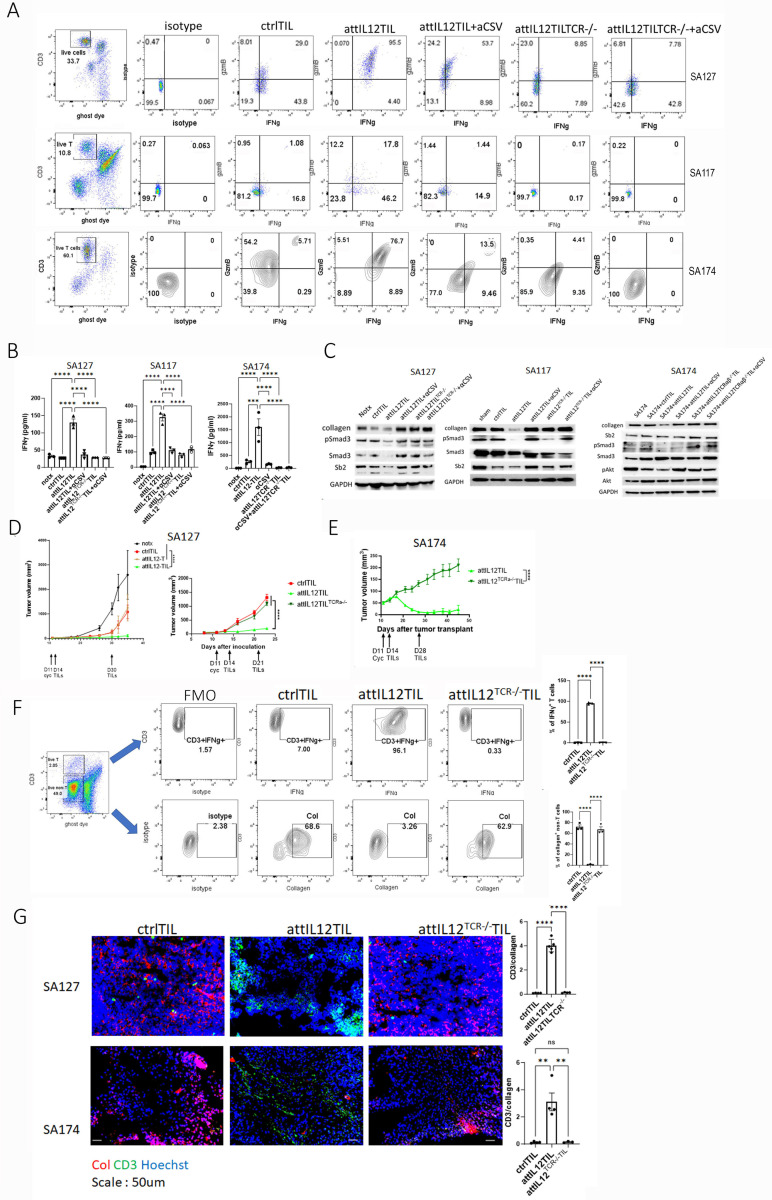
Dual signaling activation is essential for the potency of attIL12-TILs. **(A)** Flow cytometry analysis showing IFNγ and granzyme B in SA127, SA117, and SA174 tumor cells cocultured with autologous control-TILs, attIL12-TILs, or attIL12-TCRα^−/−^ TILs with or without a CSV-blocking antibody (αCSV). **(B)** ELISA analysis showing IFNγ levels in the supernatant from coculture of the indicated tumor cells and TILs. **(C)** Immunoblotting showing collagen regulation signaling in tumor cells cocultured with the indicated TILs. **(D, E)** Tumor growth in mice bearing SA127 **(D)** and SA174 **(E)** tumors treated twice with ctrl-TILs, attIL12-TILs, attIL12-T cells, or attIL12-^TRA−/−^ TILs. **(F)** Flow cytometry analysis showing IFNγ expression from TILs and collagen from SA127 tumor cells. **(G)** Representative immunofluorescence staining of human collagen (green) and CD3 (red) in SA127 and SA174 tumor sections. Scale bar: 50 μm. *P < 0.05; **P < 0.01; ***P < 0.001; ****P < 0.0001.

Unlike attIL12-T cells or unmodified TILs, attIL12-TILs induce dual signaling from 2 independent engagements with autologous sarcoma cells, one mediated by attIL12-CSV and the other by TCR-HLA. To investigate how these pathways interact to regulate IFNγ production in attIL12-TILs, we impaired CSV binding and TCR signaling separately and together. CSV signaling was blocked by the CSV-targeted antibody 84–1, as in previous studies.^20, 25, 32^ TCR signaling was abrogated by transducing a lentivirus containing a *TRA* constant regionshRNA gene construct.^33^ attIL12^TRA-/-^ TILs exhibited IL12 expression on the cell surface but no TCRα/β expression in all 3 models (Fig. S5). In all 3 autologous sarcoma PDX/TIL pairs, coculture of PDX-derived cells with autologous attIL12-TILs dramatically stimulated IFNγ and granzyme B production (Fig. 5A). Blocking CSV or knocking out *TRA* in the cocultured attIL12-TILs reduced the levels of effector cytokines produced by TILs, but shutting down both signaling pathways completely eliminated the effector cytokines (Fig. 5A). These results suggested that both CSV-attIL12 and HLA-TCR interactions between tumor cells and TILs are required to completely trigger the secretion of these effector cytokines. This dual-signaling activation hypothesis was confirmed using ELISA of IFNγ in the supernatant from the coculture of tumor cells and TILs after blockade of CSV and/or knockout of *TRA* (Fig. 5B). Thus, only attIL12-TIL/tumor cell coculture completely inhibited collagen production in the tumor cells. Omission of one of these signaling pathways (or, of course, both) failed to reduce collagen levels (Fig. S6).

Since TGFβ is highly expressed by tumor cells and triggers collagen expression,^34–36^ we next sought to determine whether attIL12-TILs interrupt TGFβ-mediated collagen expression. To decipher the mechanism by which attIL12-TILs mediate collagen reduction, we examined TGFβ downstream signaling via immunoblotting. Indeed, SPβ2 level and pSMAD3 signaling, which represent TGFβ activity, were inhibited by coculture of tumor cells with attIL12-TILs. This inhibition was associated with the reduction of collagen expression in the tumor cells after coculture (Fig. 5C), confirming that the dual signaling pathway activation by attIL12-TILs is required to inhibit collagen expression by tumor cells.

We learnt from the failure of attIL12-T cell therapy in treating ECM-rich PDX tumors that relying solely on the interaction between attIL12 on T cells and CSV on tumor cells to promote T-cell infiltration is insufficient.^25, 37^ We thus hypothesized that the interaction between attIL12-TILs and autologous tumor cells triggers additional HLA-TCR signaling between tumor cells and T cells, in turn leading to robust IFNγ expression and collagen downregulation. If this hypothesis is correct, attIL12-TIL transfer should in theory achieve much better antitumor efficacy in ECM-rich models.^38^ Indeed, our data shown in Fig. 2 confirmed this hypothesis. To test this hypothesis further, we treated SA127 tumor-bearing mice with control TILs, attIL12-T cells, or attIL12-TILs. Control TILs and attIL12-T cells, in which IFNγ induction is activated by a single signal, resulted in delayed tumor development compared to no treatment (Fig. 5D). However, attIL12-TILs, which activated both signaling pathways, both inhibited autologous tumor growth and prolonged mouse survival time (Fig. 5D, 5E). This potent tumor inhibition was impaired by *TRA* knockout, suggesting that stimulation of both IFNγ-inducing signals is needed to treat ECM-rich solid tumors (Fig. 5D, 5E).

Upon dissociating the tumors to analyze both TILs and tumor cells, we found that most of the attIL12-TILs in the tumor microenvironment produced high levels of IFNγ (Fig. 5F). Ablation of TCRα in attIL12-TILs impaired the release of IFNγ from these T cells. As a result, the high expression of collagen (Fig. 5F) in tumor cells was completely suppressed by attIL12-TIL treatment, and this effect was abrogated with *TRA* knockout (Fig. 5F). Intriguingly, control TILs could not break the collagen barrier to penetrate tumors. In contrast to the T-cell exclusion phenotype we identified in the control TIL–treated tumors, tumors treated with attIL12-TILs exhibited reduced collagen density and greater penetration of TILs into the tumor core regions (Fig. 5G). By contrast, *TRA* ablation totally impaired this attIL12-TIL–mediated collagen reduction and T-cell infiltration. Thus, all the *in vivo* data were in line with our *in vitro* discoveries that dual signaling activation by attIL12-TILs enhanced the IFNγ-dominated tumor microenvironment to suppress collagen expression.

### attIL12-TILs downregulate CCKAR, resulting in inhibition of multiple collagen expression pathways to reduce collagen

**Figure 6:**
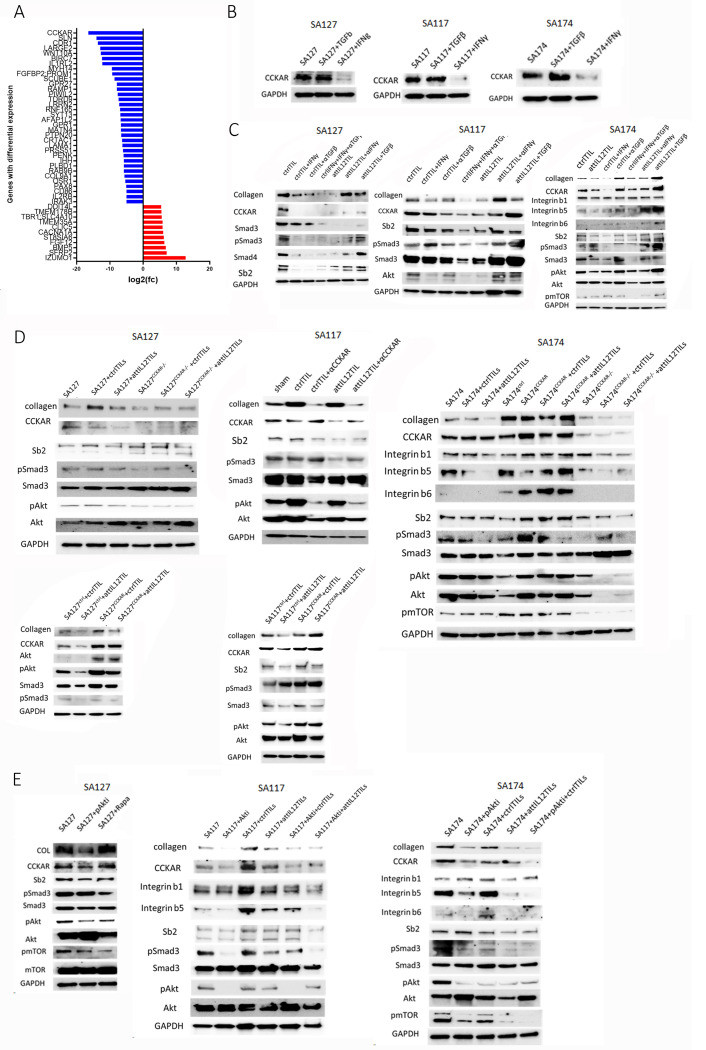
attIL12-TILs induce CCKAR downregulation to abolish its enhancer role in collagen regulation. **(A)** RNA sequencing waterfall plot showing CCKAR as the top differentially downregulated gene in attIL12-T cell treatment–sensitive osteosarcoma PDX models. **(B-E)** Immunoblots showing effects of attIL12-TILs. **(B)** CCKAR expression in the indicated tumor cells treated with TGFβ or IFNγ. **(C)** Protein expression of collagen, CCKAR, and related pathway genes in the indicated tumor cells after cocultured with the indicated TILs. TILs were treated with TGFβ, IFNγ, and/or antibodies blocking TGFβ or IFNγ. **(D)** Protein expression of collagen and related pathway genes in tumors with or without CCKAR expression and cocultured with control or attIL12-TILs. CCKAR was inactivated either via shRNA transduction (CCKAR^−/−^) or a blocking antibody (αCCKAR). **(E)** Expression of collagen, CCKAR, and related pathway genes in the indicated tumor cells cocultured with control or attIL12-TILs treated with the AKT inhibitor (Akti) MK-2206.

In our previous attIL12-T cell therapy study, we compared the transcriptome profiles of 3 models that were responsive to the treatment with those of 3 other models that failed to respond.^25^ The RNA sequencing data showed that the most highly downregulated gene in responsive tumors was *CCKAR* (Fig. 6A). *CCKAR* encodes a G-protein coupled receptor that binds to the cholecystokinin family. However, the exact pathways by which CCKAR contributes to collagen expression in tumors remained unknown.

Given our finding that attIL12-TIL–induced collagen reduction depended on both IFNγ elevation and TGFβ signaling inhibition (Fig. 5F), we investigated the impact of CCKAR expression on IFNγ and TGFβ levels. We found that CCKAR expression was dramatically reduced by IFNγ (Fig. 6B). To investigate how IFNγ and TGFβ affect CCKAR and collagen expression in the presence of TILs, we cocultured tumor cells with autologous control TILs or attIL12-TILs with or without additional IFNγ, TGFβ, or their neutralizing antibodies (Fig. 6C). As shown in Fig. S6, attIL12-TIL coculture dramatically decreased collagen expression compared to control TIL coculture. Either adding IFNγ or blocking TGFβ reduced both collagen and CCKAR expression, whereas adding TGFβ or blocking IFNγ increased collagen and CCKAR expression (Fig. 6C), evidence of a positive correlation between CCKAR and collagen expression.

TGFβ is known to stimulate pSMAD3 signaling for collagen regulation, and AKT activation has crosstalk with pSMAD3 signaling in collagen regulation.^39–41^ Therefore, we also assessed pSMAD3, SPβ2, and pAKT levels (Fig. 6C). To further decipher how CCKAR regulates collagen expression, we impaired CCKAR via a blocking antibody or viral transduction of *CCKAR* shRNAs (Fig. S7A) prior to coculture with control or attIL12-TILs (Fig. 6D). CCKAR impairment alone in the control TIL coculture was sufficient to cause collagen reduction and pAKT downregulation (Fig. 6D), suggesting that decreased CCKAR expression alone may downregulate collagen production through the pAKT pathway. This conclusion was further supported by the results from CCKAR-overexpressing tumor cells (Fig. S7B) cocultured with control or attIL12-TILs (Fig. 6D). High CCKAR expression in the tumor cells amplified pAKT-induced collagen expression even after attIL12-TIL coculture, suggesting that under low levels of TGFβ and pSMAD3 activation, constitutive expression of CCKAR functions as a robust inducer of AKT signaling and in turn promotes collagen expression in sarcoma cells.

**Figure 7:**
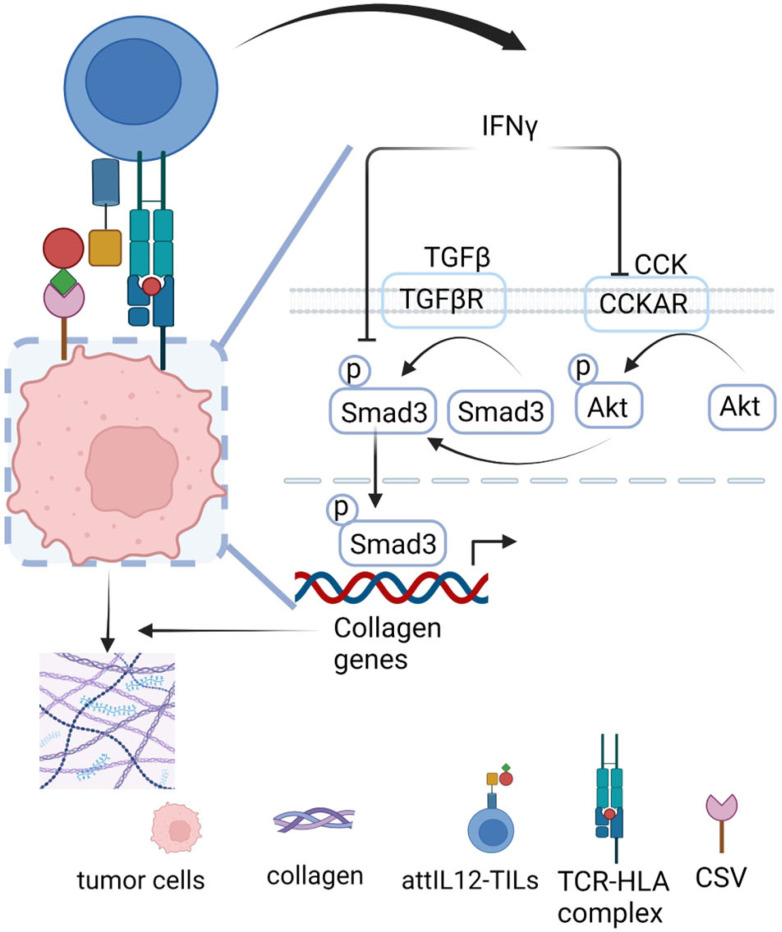
Model of the molecular mechanisms underlying collagen suppression by attIL12-TILs. The interaction of attIL12-TILs and autologous tumor cells induces IFNγ through simultaneous activation of attIL12 binding to CSV and HLA-TCR. The IFNγ-dominant tumor environment inhibits TGFβ-dependent SMAD3 activation and TGFβ-independent CCKAR signaling–mediated collagen expression to make collagen-rich tumors accessible to attIL12-TILs.

To validate the role of AKT phosphorylation in collagen regulation, we used the AKT inhibitor MK-2206 during the coculture with control or attIL12-TILs (Fig. 6E). Inhibition of pAKT inhibited collagen expression in tumor cells cultured alone or cocultured with control TILs or attIL12-TILs, suggesting that attIL12-TIL coculture not only abolished TGFβ-induced SMAD3 signaling but also disabled TGFβ-independent CCKAR-AKT activation to suppress collagen expression. Collectively, our results clearly demonstrated that attIL12-TIL–induced IFNγ elevation impaired both TGFβ-SMAD3 and CCKAR-pAKT activation–mediated collagen expression. These mechanisms explain how attIL12-TILs are able to destroy the collagen barrier and enhance the efficacy of TIL therapy (Fig. 7).

## Discussion

Here, we discovered that attIL12 modification of TILs synergizes IFNγ production in tumors (but not in peripheral tissues), enhances TIL infiltration into autologous collagen-rich PDX tumors, and inhibits tumor progression. This potent enhancement requires simultaneous attIL12 binding to CSV and TCR activation by engaging autologous tumor cells. The robust induction of IFNγ suppressed both TGFβ-dependent SMAD3 activation and CCKAR-AKT signaling–mediated collagen expression in the tumor microenvironment. The diminished collagen expression induced a robust infiltration of TILs to attack tumor cells.

Our strategy overcomes several known and potential challenges to TIL therapy. One potential challenge for TIL therapy is that sarcomas are generally not considered “hot tumors,” meaning they lack infiltration by effector immune cells. However, studies in patient samples confirmed that TILs are present in the majority of localized osteosarcomas^3^ and can be successfully expanded from undifferentiated sarcoma tumors.^42^ In this regard, we have used our established platform^43, 44^ in which “off-the-shelf” feeder cells developed from artificial antigen-presenting cells expressing T-cell costimulatory molecules are used for TIL expansion from sarcoma clinical samples. However, studies have shown that TILs can lose their ability to penetrate tumors after *ex vivo* expansion regardless of the technique used.^45^ One reason for this is that during the expansion process, TILs are often stimulated with high levels of IL2, which can cause them to differentiate into cells with effector phenotypes.^46^ These effector cells are better at killing cancer cells but may have reduced tumor-infiltrating ability. Another factor is that TILs may undergo genetic changes during the expansion process so that they differentiate into clusters with different TCR repertoires,^47^ which can affect their ability to recognize and infiltrate tumors. Additionally, TILs may become exhausted or senescent during the expansion process, which may also reduce their tumor-infiltrating ability.^48^ We have shown, from a different perspective, that the impairment of TIL infiltration in sarcomas is mainly due to their rich collagen barrier, which hampers T cells from reaching tumors. Our attIL12-TILs not only exhibit cytolytic activity against tumor cells *in vitro*, but more importantly destroy the stiff collagen layer around tumor cells *in vivo* to allow large numbers of effector TILs to infiltrate the tumors. This approach therefore modulates the sarcoma tumor environment to make it more favorable for TIL adoptive transfer therapy. This approach is highly clinically relevant because high-grade sarcoma is associated with high-density collagen deposition.

TGFβ functions as a protumor factor to promote primary tumor growth and dissemination of metastases in sarcomas, and TGFβ level has been associated with advanced and metastatic osteosarcoma in clinical samples.^49, 50^ In the sarcoma microenvironment, TGFβ is mainly expressed by tumor cells and stromal cells (e.g., CAFs, endothelial cells) and triggers ECM factor expression. This TGFβ-dominated context in sarcomas leads to aberrant collagen gene expression.

It is well understood that TGFβ and IFNγ counteract each others’ signaling. On one hand, as an immunosuppressive cytokine, TGFβ suppresses cytotoxic T cells to produce effector molecules, including IFNγ, and downstream STAT1 activation during tumor cell killing.^51, 52^ On the other hand, a high level of IFNγ downregulates ECM factors, especially the integrins, which play crucial roles in TGFβ activation. The inactive TGFβ is bound to latent-associated protein (LAP) on the cell surface. To initiate TGFβ signaling, inactive TGFβ needs to be released from LAP so that it can interact with its receptor. This release requires integrin-applied force.^53–55^ Although IFNγ induction may not reduce the total level of TGFβ in the tumor microenvironment, it does attenuate TGFβ maturation and therefore interrupts collagen expression. Moreover, IFNγ stimulates JAK1-STAT1 signaling to induce SMAD7, which antagonizes TGFβ downstream SMAD3 phosphorylation, nuclear translocation, and activation of responsive genes (such as collagen genes).^56^ Therefore, we hypothesized that shifting the sarcoma microenvironment from a TGFβ-dominated one to an IFNγ-dominated one would overcome the collagen barrier that blocks T-cell infiltration. This concept was demonstrated by the addition of recombinant human IFNγ, which abolished collagen expression in 3 independent sarcoma models. However, it is not feasible to provide constitutively high levels of IFNγ by using the recombinant protein, so the challenge was to find an effective and safe approach that maintains IFNγ production in tumors. Remarkably, the interaction between attIL12-TILs and autologous tumor cells induced robust levels of IFNγ *in vitro* and *in vivo* to suppress TGFβ-induced collagen overexpression and, in turn, to enhance TIL infiltration into tumors.

CCKAR first came to our attention in our previous total RNA sequencing results comparing the transcriptomes of attIL12-T cell–responsive and –resistant osteosarcoma PDX models^25^; CCKAR was the most significantly reduced gene in the responsive tumors. Unlike TGFβ, CCKAR’s role in tumor collagen expression is not well understood, but our RNA sequencing results found that CCKAR is highly overexpressed in ECM-rich resistant PDX models. Studies have also shown that CCKAR expression is downregulated by IFNγ.^19^ Here, we observed CCKAR downregulation by IFNγ in all 3 sarcoma PDX cell lines we generated. In these sarcoma cell lines, CCKAR regulates collagen expression, which can be markedly decreased by IFNγ elevation. We established *CCKAR*-overexpressing and *CCKAR*-knockout stable cell lines to demonstrate that CCKAR can induce collagen expression via AKT activation and impact pSMAD3 signaling, suggesting its pivotal role in collagen regulation. Altogether, these results emphasized the importance of the transition from a TGFβ- to an IFNγ-dominated tumor microenvironment, which shuts down multiple pathways through which TGFβ and CCKAR signaling limit collagen expression.

The next question was how attIL12-TILs induce greater IFNγ production than attIL12-T or control TILs when interacting with tumor cells. We previously administered attIL12-T cells for treatment of osteosarcoma PDX tumors and found that the rich ECM structure throughout the entire tumor was impossible to penetrate.^25^ Although we demonstrated that the interaction between attIL12 from attIL12-T cells and CSV from tumor cells stimulates IFNγ production,^23^ obviously this IFNγ level was not enough to overcome the total collagen barrier in ECM-rich sarcoma tumors. One major limitation of our previous attIL12-T cell therapy approach was the lack of tumor-specific T cells (such as TILs and TCR-T cells) to trigger TCR activation. This limitation led to unknown responses to autologous immune cells in preclinical studies and poor prediction of outcomes in human trials.^57^ In this study, the reason for using autologous PDX/TIL pairs was to stimulate a tumor-specific TIL repertoire which synergized with attIL12-CSV binding to maximize IFNγ production in the sarcoma tumor environment. We validated this notion by blocking CSV binding, impairing TCRα via shRNA transduction, or both. We observed that abrogation of either signaling pathway dramatically compromised collagen reduction, suggesting that the dual signaling pathways supporting IFNγ elevation were the key to the success of attIL12-TIL treatment. Besides TIL therapy, attIL12 may synergize with other T-cell therapy strategies that induce robust IFNγ production (e.g., TCR-T or CAR-T cell therapy) for solid tumor treatment, suggesting broader applications for attIL12 engineered T-cell treatments.

## Materials and Methods

### Animal studies and tumor models

Six- to eight-week-old C.B-17SC *scid*^−/−^ mice of both sexes were purchased from The Jackson Laboratory. The mouse care and handling procedures were approved by the Institutional Animal Care and Use Committee of The University of Texas MD Anderson Cancer Center.

To generate PDX tumors in mice, patient-derived SA127, SA117, and SA174 sarcoma tumors (generously provided by Dr. Richard Gorlick, the Pediatric Preclinical Testing Consortium, The University of Texas MD Anderson Cancer Center) were implanted subcutaneously into C.B-17SC *scid*^−/−^ mice. When tumors reached 6~8 mm in diameter, mice were preconditioned with cyclophosphamide (Baxter Healthcare), followed by 2 infusions of 2 × 10^6^ T cells 14 days apart. Next, 10,000 U of human IL2 was injected subcutaneously for the first 3 days following TIL transfer and twice weekly afterwards for 3 weeks. Tumors were measured with calipers twice weekly after implantation. Tumor volume was calculated by the formula V = (π /8) × (a b^2^), where V = tumor volume in cubic centimeters, a = maximum tumor diameter, and b = diameter at 90° to a.

### Cell culture

SA127, SA117, and SA174 PDX tumors were harvested, and necrotic tissues were removed. Tumor tissues were minced into 0.2-cm^3^ pieces and digested in 10 mg collagenase II in 20 mL Dulbecco’s modified Eagle’s medium (DMEM) at 37 °C for 1 h with rotation. The cell mixtures were filtered with a strainer (100 μm pore size) to remove remaining tissues and washed with 20 mL DMEM. Cell suspensions were spun down at 600 *g* for 10 minutes and washed with 10 mL DMEM 3 times. After the final wash, cell pellets were resuspended in 10 mL cell culture medium and placed into a 6-well plate. Established PDX tumors and cell lines were authenticated using short tandem repeat profiling analysis to ensure that they exhibited the same features as the original samples.

SA127 human undifferentiated sarcoma cells were cultured in DMEM containing 10% fetal bovine serum (FBS) supplemented with antibiotics and nonessential amino acid solution, and maintained in an incubator at 5% CO_2_ and 37 °C. SA117 and SA174 osteosarcoma cells were cultured in DMEM containing high glucose and 10% FBS supplemented with antibiotics and nonessential amino acid solution, and were maintained in an incubator at 5% CO_2_ and 37 °C. The tumor cell lines were characterized by DNA fingerprinting at MD Anderson Cancer Center's Characterized Cell Line Core Facility within 6 months of initiating the experiments and treated with a mycoplasma removal agent from Bio-Rad.

### Human TIL isolation and expansion

K562 artificial antigen-presenting cells (AaPCs) expressing CD64, CD86, and CD137L modified to express a membrane-bound IL15/IL15Rα fusion protein ^58,59^ were used to expand TILs. K562 AaPCs were cultured in VueLife bags and/or the WAVE Bioreactor ^60^ and phenotyped to validate expression of the introduced transgenes/costimulatory molecules. Anti-CD3 antibodies (OKT3, Orthoclone) were loaded onto the cell surface of the K562 AaPCs via the CD64/FcγR (OKT3-K562 AaPC), and then the cells were irradiated at 100 Gy to inhibit proliferation and frozen in aliquots for later use. ^60^ Tumor tissue was enzymatically dissociated in media (10% RPMI) to create a single-cell suspension. Isolated cells were cocultured with OKT3-K562 AaPCs and exogenous cytokines (IL2 and IL21) in a 7-day stimulation cycle for 4–5 weeks. Cells were expanded at the end of each stimulation cycle.

### Plasmid constructs[AN1]

Human IL12 subunit P35 with and without a transmembrane domain and subunit P40 with and without a tumor-targeted peptide were synthesized by Vector Builder and cloned into a third-generation self-inactivating lentiviral expression vector (Vector Builder) under a murine stem cell virus and modified cytomegalovirus promoter.

Human CCKAR: pLV-CMV-mCherry-MSCV>hCCKAR[NM_000730.3]

hCCKAR [NM_000730.3] was cloned under an MSCV retrovirus promoter on Vector Builder’s mammalian expression lentiviral vector.

*CCKAR* shRNA: pLV-EGFP:T2A:Puro-U6>hCCKAR[shRNA)

VectorBuilder’s shRNA (3+1) virus packaging services include cloning and packaging 3 custom shRNA viruses targeting hCCKAR and one scrambled control virus.

*hCCKAR*[shRNA#1] Target Sequence: ACCACCAGCAGCGGCAAATAT

*hCCKAR*[shRNA#2] Target Sequence:TAACAACCAGACCGCGAATAT

*hCCKAR*[shRNA#3] Target Sequence: CTCTTGTACTCCTTGATATTC

*TRA* shRNA: pLV-U6 >{hTCR-gRNA#1}-EFS>hCas9: hTCR-gRNA#1 targeting sequence:

TCTCTCAGCTGGTACACGGC were cloned under a U6 promoter in Vector Builder’s mammalian CRISPR lentiviral vector. The scrambled control was cloned in the same vector.

All genes were constructed and lentivirus manufactured by Vector Builder.

### Generation of lentivirus

High-titer replication-defective lentiviral vectors were produced and concentrated by Vector Builder based on their procedures.

### Human TIL lentiviral transduction

The lentiviral supernatant was first centrifuged at 2000 *g* for 1.5 h on retronectin (Takara)-coated non-tissue culture–treated plates. TILs were then plated and centrifuged at 600 *g* for 20 minutes and incubated at 37 °C. After 3 days, the medium was changed to 45% RPMI-1640 and 45% Click’s medium containing 10% FBS and supplemented with recombinant human IL2 (50 U/mL) and IL21 (10 ng/mL).

### Total RNA sequencing

The harvested osteosarcoma PDX tumor samples were processed and analyzed by LC Sciences. Briefly, total RNA was extracted using Trizol reagent (Thermo Fisher) following the manufacturer's procedure. The total RNA quantity and purity were analyzed using a Bioanalyzer 2100 system and RNA 6000 Nano LabChip, and high-quality RNA samples with RNA integrity number (RIN) >7.0 were used to construct the sequencing library. After total RNA was extracted, mRNA was purified from 5 μg total RNA using Dynabeads Oligo (dT) (Thermo Fisher) with 2 rounds of purification. The 2 × 150-bp paired-end sequencing (PE150) was performed on an Illumina Novaseq 6000 system following the vendor's recommended protocol. All samples were aligned to the human reference genome using the HISAT2 (https://daehwankimlab.github.io/hisat2/,version:hisat2-2.0.4) package. Analysis of differentially expressed genes (DEGs) was performed by DESeq2 software for comparisons of 2 groups and by edgeR for comparisons of 2 samples. Genes with a false discovery rate below 0.05 and absolute fold change ≥2 were considered DEGs.

DEGs were then subjected to analysis of enrichment of Gene Ontology (GO) functions and Kyoto Encyclopedia of Genes and Genomes (KEGG) pathways. For the GO enrichment analysis, all DEGs were mapped to GO terms in the Gene Ontology database (http://www.geneontology.org/), gene numbers were calculated for every term, and GO terms significantly enriched in DEGs compared to the reference genome were defined by hypergeometric test. KEGG pathway enrichment analysis identified significantly enriched metabolic pathways or signal transduction pathways in DEGs compared with the whole genome background (https://www.kegg.jp/kegg/).

### National Cancer Institute GDC Data Portal

*COL1A1*, *COL2A1*, and *COL6A1* gene expression in tumors from sarcoma patients and patients’ overall survival time were determined using the sarcoma dataset from The Cancer Genome Atlas data portal TCGA-SARC (https://portal.gdc.cancer.gov/projects/TCGA-SARC). We stratified patients by combined collagen gene expression. The top 20% were considered the pan-collagen high expression group, and bottom 20% were considered the pan-collagen low expression group. Kaplan-Meier curves were used to compare the overall survival time between the 2 groups.

### cBioPortal for cancer genomics

*TGFB1* and *COL1A1* gene expression data were obtained from The Cancer Genome Atlas portal (https://www.cbioportal.org/). To identify relationships between the expression levels of these genes, Pearson correlation coefficients were calculated using the R statistical computing package.

### Immunoblotting

Frozen tissue samples were smashed before being homogenized using a minibead beater with 5 to 8 silicone beads (BioSpec Products) in 0.4 mL of ice-cold radioimmunoprecipitation assay lysis buffer. The homogenized tumor cells were then subjected to lysis with this buffer. The protein extracts were separated from the tissue residues by centrifugation at the maximum speed for 20 minutes at 4 °C. Forty-microgram samples of total protein were fractionated by 10% sodium dodecyl sulfate- polyacrylamide gel electrophoresis and transferred to nitrocellulose membranes using a Trans-Blot Turbo transfer system (Bio-Rad). The membranes were blotted with different primary and secondary antibodies (see Antibody list) to detect the proteins of interest.

### Immunohistochemistry and immunofluorescence staining

Frozen tumor sections were sequentially fixed with cold acetone, acetone plus chloroform (1:1), and acetone. Paraffin-embedded sections were deparaffinized and heated in antigen retrieval buffer. Tissue sections were blocked with 3% H_2_O_2_ in distilled water for 20 minutes and then in blocking buffer (5% normal horse serum and 1% normal goat serum in PBS). Slides were incubated with primary antibodies (see Antibody list) overnight at 4 °C and secondary antibodies (see Antibody list) for 1 h at room temperature. For immunohistochemistry staining, the secondary antibody was biotin conjugated, the sections were treated with ABC reagent (Vector Labs), and the nuclei were counterstained with hematoxylin (Sigma-Aldrich). Tumor sections were mounted with Cytoseal mounting medium (Life Technologies). Quantifications of immunohistochemistry images were assessed by examining 3 randomly selected low-power fields per slide. For immunofluorescence staining, tumor sections were mounted in an antifade fluorescence mounting medium with 4′,6-diamidino-2-phenylindole. Slides were visualized under a Nikon Eclipse Ti fluorescence microscope.

### Enzyme-linked immunosorbent assay

Culture medium was collected from coculture experiments at 1 mL medium/10^6^ T cells. The level of IFNγ was measured by using ELISA Ready-SET-Go! kits (eBioscience) or ELISA Kit Picokine (Boster Bio).

### Flow cytometry

Cells were sequentially incubated with primary and secondary antibodies for 30 minutes each at 4°C. Stained cells were analyzed using an Attune acoustic focusing cytometer (Applied Biosystems) or a BD LSR-Fortessa cell analyzer (BD Biosciences). Flow cytometry data were analyzed using the FlowJo software program (FlowJo, LLC).

### Tumor-cell dissociation

Tumors were minced into 2-mm fragments, placed in 5 mL of dissociation buffer (RPMI-1640 medium with 100 U/mL collagenase type IV and 100 U/mL DNase I), and incubated at 37 °C while shaking at 120 rpm for 30 minutes to 1 h. The released cells were filtered with 70-μm strainers and centrifuged at 600 *g* for 5 minutes, followed by red blood cell lysis. Cells were then resuspended in fluorescence-activated cell sorting solution containing 2% FBS. Single-tumor-cell suspensions were obtained after CD45 depletion using an EasySep Human CD45 Depletion Kit (Stem Cell Technologies).

### Statistical analysis

The directly measured outcomes were analyzed using a 2-sided Student *t*-test to compare 2 treatment groups or 1-way analysis of variance to compare more than 2 treatment groups. The statistical analyses were conducted using GraphPad Prism 8 software. All data values represent replicates and are shown as mean ± SEM. Significance levels were defined as *P < 0.05; **P < 0.01; ***P < 0.001; ****P < 0.0001. All experiments were repeated at least 3 times.

## Supplementary Material

Supplement 1
