# Supplementary material for: Collagen-disruptive cell therapy: adoptive transfer of membrane-anchored, tumor cell surface vimentin-targeted interleukin 12–armed TILs suppress collagen expression to boost deep T-cell infiltration via dual signaling activation and significant CCKAR reduction
Source: Res Sq. 2024 Oct 29:rs.3.rs-5104493. Preprint. [Version 1] doi: 10.21203/rs.3.rs-5104493/v1 (PMC11581118; doi:10.21203/rs.3.rs-5104493/v1)
Supplement: Supplement 1 [file NIHPPRS5104493V1-supplement-1.pdf]

Supplementary Files

This is a list of supplementary files associated with this preprint. Click to download.

- SupplementalFiguresANed.docx
